# Supplementary material for: Improved Oral Health Status Is Associated with a Lower Risk of Venous Thromboembolism: A Nationwide Cohort Study
Source: J Pers Med. 2022 Dec 22;13(1):20. doi: 10.3390/jpm13010020 (PMC9863073; doi:10.3390/jpm13010020)
Supplement: Supplementary file 1 [file jpm-13-00020-s001.zip › jpm-2066317-supplementary.pdf]

Supplementary Table S1. Risk factors for the occurrence of venous thromboembolism.

Variable	Crude HR (95% CI)	p-value	Adjusted HR (95% CI)	p-value
Age, years				
<65	1 (reference)		1 (reference)	
≥65	4.52 (4.40, 4.63)	<.001	2.72 (2.64, 2.79)	<.001
Sex				
Male	1 (reference)		1 (reference)	
Female	1.32 (1.30, 1.35)	<.001	1.12 (1.09, 1.15)	<.001
Body mass index (kg/m^2)	1.00 (1.00, 1.00)	<.001	1.00 (1.00, 1.00)	<.001
Household income				
Q1, lowest	1 (reference)		1 (reference)	
Q2	0.82 (0.80, 0.84)	<.001	0.91 (0.89, 0.94)	<.001
Q3	0.85 (0.83, 0.88)	<.001	0.91 (0.89, 0.94)	<.001
Q4, highest	1.01 (0.98, 1.04)	0.667	0.95 (0.92, 0.99)	0.006
Smoking				
Never	1 (reference)		1 (reference)	
Former	0.80 (0.78, 0.83)	<.001	0.94 (0.90, 0.97)	0.001
Current	0.74 (0.72, 0.76)	<.001	0.96 (0.93, 0.98)	0.002
Alcohol consumption (days/week)				
<1	1 (reference)		1 (reference)	
1-4	0.78 (0.76, 0.80)	<.001	0.93 (0.91, 0.96)	<.001
≥5	1.80 (1.71, 1.88)	<.001	1.56 (1.49, 1.64)	<.001
Regular physical activity (days/week)				
<1	1 (reference)		1 (reference)	

1-4	0.76 (0.74, 0.77)	<.001	0.86 (0.85, 0.88)	<.001
≥5	1.31 (1.26, 1.35)	<.001	1.13 (1.09, 1.17)	<.001
Comorbidities				
Hypertension	2.87 (2.82, 2.93)	<.001	2.11 (2.06, 2.15)	<.001
Diabetes mellitus	2.47 (2.40, 2.53)	<.001	1.57 (1.53, 1.62)	<.001
Dyslipidemia	1.88 (1.83, 1.92)	<.001	1.33 (1.29, 1.36)	<.001
Atrial fibrillation	5.10 (4.52, 5.74)	<.001	2.12 (1.88, 2.39)	<.001
Cancer	2.60 (2.43, 2.79)	<.001	1.81 (1.69, 1.94)	<.001
Renal disease	3.38 (3.12, 3.66)	<.001	1.30 (1.20, 1.41)	<.001
Antiphospholipid syndrome	2.62 (2.18, 3.14)	<.001	1.44 (1.20, 1.72)	<.001
Osteoporotic fracture	2.58 (2.37, 2.81)	<.001	1.45 (1.33, 1.58)	<.001
Oral health status				
Periodontitis				
No	1 (reference)		1 (reference)	
Yes	1.47 (1.40, 1.54)	<.001	1.21 (1.15, 1.28)	<.001
Number of missing teeth				
0	1 (reference)		1 (reference)	
1-7	1.63 (1.60, 1.67)	<.001	1.38 (1.35, 1.42)	<.001
8-14	3.03 (2.83, 3.25)	<.001	1.59 (1.49, 1.71)	<.001
≥15	3.82 (3.54, 4.13)	<.001	1.58 (1.46, 1.71)	<.001
Oral hygiene behaviors				
Frequency of tooth brushing (times/day)				
0-1	1 (reference)		1 (reference)	

2	0.77 (0.75, 0.79)	<.001	0.87 (0.84, 0.89)	<.001
≥3	0.51 (0.50, 0.53)	<.001	0.67 (0.65, 0.69)	<.001
Dental visit for any reason				
No	1 (reference)		1 (reference)	
Yes	1.04 (1.02, 1.06)	0.001	1.05 (1.03, 1.07)	<.001
Dental scaling				
No	1 (reference)		1 (reference)	
Yes	0.87 (0.85, 0.89)	<.001	0.95 (0.93, 0.98)	<.001

Multivariable model was sex, age, body mass index, income levels, smoking, alcohol consumption, regular physical activity, hypertension, diabetes mellitus, dyslipidemia, atrial fibrillation, cancer, renal disease, antiphospholipid syndrome, and osteoporotic fracture.

Q, Quartile; HR, hazard ratio; CI, confidence interval.

Supplementary Table S2. The subgroup analysis regarding periodontitis and venous thromboembolism in association with demographics or comorbidities.

Variable	Adjusted HR (95% CI)	p-value for interaction effect
Age, years		<.001
<65	1.28 (1.21, 1.36)	
≥65	0.97 (0.87, 1.07)	
Sex		0.101
Male	1.22 (1.14, 1.30)	
Female	1.18 (1.09, 1.28)	
Body mass index (kg/m^2)		0.103
<25	1.26 (1.18, 1.35)	
≥25	1.11 (1.03, 1.20)	
Household income		0.572
Q1, lowest	1.17 (1.07, 1.28)	
Q2	1.26 (1.15, 1.37)	
Q3	1.21 (1.09, 1.34)	
Q4, highest	1.19 (1.03, 1.38)	
Smoking		0.089
Never	1.20 (1.13, 1.28)	
Former	1.06 (0.90, 1.25)	
Current	1.28 (1.17, 1.40)	
Alcohol consumption (days/week)		0.071

<1	1.23 (1.16, 1.31)	
1-4	1.22 (1.10, 1.35)	
≥5	0.99 (0.81, 1.19)	
Regular physical activity (days/week)		0.894
<1	1.23 (1.15, 1.31)	
1-4	1.17 (1.07, 1.29)	
≥5	1.24 (1.07, 1.44)	
Comorbidities		
Hypertension		0.142
No	1.25 (1.17, 1.34)	
Yes	1.15 (1.07, 1.24)	
Diabetes mellitus		0.061
No	1.25 (1.18, 1.32)	
Yes	1.06 (0.95, 1.19)	
Dyslipidemia		0.059
No	1.23 (1.16, 1.30)	
Yes	1.12 (1.00, 1.25)	
Atrial fibrillation		0.548
No	1.21 (1.15, 1.28)	
Yes	1.30 (0.77, 2.20)	
Cancer		0.946
No	1.21 (1.15, 1.27)	

Yes	1.26 (0.92, 1.74)	
Renal disease		0.268
No	1.22 (1.16, 1.28)	
Yes	0.78 (0.50, 1.24)	
Antiphospholipid syndrome		0.819
No	1.21 (1.15, 1.28)	
Yes	1.40 (0.68, 2.91)	
Osteoporotic fracture		0.053
No	1.40 (0.68, 2.91)	
Yes	0.80 (0.50, 1.26)	

Multivariable model was sex, age, body mass index, income levels, smoking, alcohol consumption, regular physical activity, hypertension, diabetes mellitus, dyslipidemia, atrial fibrillation, cancer, renal disease, antiphospholipid syndrome, and osteoporotic fracture.
HR, hazard ratio; CI, confidence interval.

Supplementary Table S3. The risk for occurrence of deep vein thrombosis according to oral health status and oral hygiene behaviors.

	Number of participants	Number of events	Event rate (%) (95% CI)	Person-years	Incidence rate (per 1000 person-years)	Adjusted HR (95% CI)	p-value
Oral health status							
Periodontitis							
No	2184950	14632	0.67 (0.66, 0.68)	35775531.60	0.41	1 (reference)	
Yes	61720	543	0.88 (0.81, 0.95)	991706.30	0.55	1.10 (1.01, 1.19)	0.037
Number of missing teeth							
0	1847223	11125	0.60 (0.59, 0.61)	30400383.65	0.37	1 (reference)	
1-7	365761	3509	0.96 (0.93, 0.99)	5896826.56	0.60	1.40 (1.35, 1.46)	<.001
8-14	19586	308	1.57 (1.40, 1.75)	286214.25	1.08	1.55 (1.38, 1.74)	<.001
≥15	14100	233	1.65 (1.44, 1.86)	183813.44	1.27	1.45 (1.27, 1.66)	<.001
Oral hygiene behaviors							
Frequency of tooth brushing (times/day)							
0-1	279192	2645	0.95 (0.91, 0.98)	4437065.93	0.60	1 (reference)	
2	1041889	7869	0.76 (0.74, 0.77)	17043114.76	0.46	0.86 (0.82, 0.90)	<.001
≥3	925589	4661	0.50 (0.49, 0.52)	15287057.21	0.31	0.65 (0.62, 0.69)	<.001
Dental visit for any reason							
No	1348012	8910	0.66 (0.65, 0.67)	22004381.20	0.41	1 (reference)	
Yes	898658	6265	0.70 (0.68, 0.71)	14762856.71	0.42	1.06 (1.03, 1.10)	<.001
Dental scaling							
No	1732641	12122	0.70 (0.69, 0.71)	28281542.41	0.43	1 (reference)	

Yes	514029	3053	0.59 (0.57, 0.62)	8485695.49	0.36	0.93 (0.90, 0.97)	0.001

Multivariable model was sex, age, body mass index, income levels, smoking, alcohol consumption, regular physical activity, hypertension, diabetes mellitus, dyslipidemia, atrial fibrillation, cancer, renal disease, antiphospholipid syndrome, osteoporotic fracture.

CI, confidence interval; HR, hazard ratio.

Supplementary Table S4. The risk for occurrence of pulmonary thromboembolism according to oral health status and oral hygiene behaviors.

	Number of participants	Number of events	Event rate (%) (95% CI)	Person-years	Incidence rate (per 1000 person-years)	Adjusted HR (95% CI)	p-value
Oral health status							
Periodontitis							
No	2184950	10143	0.46 (0.46, 0.47)	35808604.77	0.28	1 (reference)	
Yes	61720	499	0.81 (0.74, 0.88)	992193.68	0.50	1.37 (1.25, 1.50)	<.001
Number of missing teeth							
0	1847223	7576	0.41 (0.40, 0.42)	30426352.97	0.25	1 (reference)	
1-7	365761	2519	0.69 (0.66, 0.72)	5903858.54	0.43	1.42 (1.36, 1.49)	<.001
8-14	19586	305	1.56 (1.38, 1.73)	286626.69	1.06	1.94 (1.72, 2.18)	<.001
≥15	14100	242	1.72 (1.50, 1.93)	183960.25	1.32	1.81 (1.59, 2.07)	<.001
Oral hygiene behaviors							
Frequency of tooth brushing (times/day)							
0-1	279192	2066	0.74 (0.71, 0.77)	4442221.08	0.47	1 (reference)	
2	1041889	5549	0.53 (0.52, 0.55)	17059842.02	0.33	0.82 (0.78, 0.86)	<.001
≥3	925589	3027	0.33 (0.32, 0.34)	15298735.36	0.20	0.60 (0.57, 0.64)	<.001
Dental visit for any reason							
No	1348012	6341	0.47 (0.46, 0.48)	22023908.97	0.29	1 (reference)	
Yes	898658	4301	0.48 (0.46, 0.49)	14776889.48	0.29	1.03 (0.99, 1.07)	0.125
Dental scaling							
No	1732641	8547	0.49 (0.48, 0.50)	28308118.47	0.30	1 (reference)	

Yes	514029	2095	0.41 (0.39, 0.43)	8492679.99	0.25	0.94 (0.89, 0.98)	0.007

Multivariable model was sex, age, body mass index, income levels, smoking, alcohol consumption, regular physical activity, hypertension, diabetes mellitus, dyslipidemia, atrial fibrillation, cancer, renal disease, antiphospholipid syndrome, osteoporotic fracture.

CI, confidence interval; HR, hazard ratio.

Supplementary Table S5. The risk for occurrence of intra-abdominal thromboembolism according to oral health status and oral hygiene behaviors.

	Number of participants	Number of events	Event rate (%) (95% CI)	Person-years	Incidence rate (per 1000 person-years)	Adjusted HR (95% CI)	p-value
Oral health status							
Periodontitis							
No	2184950	17605	0.81 (0.79, 0.82)	35734066.17	0.49	1 (reference)	
Yes	61720	733	1.19 (1.10, 1.27)	989448.57	0.74	1.21 (1.12, 1.30)	<.001
Number of missing teeth							
0	1847223	13396	0.73 (0.71, 0.74)	30367947.51	0.44	1 (reference)	
1-7	365761	4296	1.17 (1.14, 1.21)	5886612.83	0.73	1.37 (1.32, 1.42)	<.001
8-14	19586	353	1.80 (1.61, 1.99)	285669.56	1.24	1.46 (1.31, 1.62)	<.001
≥15	14100	293	2.08 (1.84, 2.32)	183284.85	1.60	1.57 (1.39, 1.76)	<.001
Oral hygiene behaviors							
Frequency of tooth brushing (times/day)							
0-1	279192	3117	1.12 (1.08, 1.16)	4430493.11	0.70	1 (reference)	
2	1041889	9466	0.91 (0.89, 0.93)	17020147.84	0.56	0.90 (0.86, 0.94)	<.001
≥3	925589	5755	0.62 (0.61, 0.64)	15272873.80	0.38	0.72 (0.69, 0.75)	<.001
Dental visit for any reason							
No	1348012	10833	0.80 (0.79, 0.82)	21979193.90	0.49	1 (reference)	
Yes	898658	7505	0.84 (0.82, 0.85)	14744320.85	0.51	1.04 (1.01, 1.07)	0.013
Dental scaling							
No	1732641	14465	0.83 (0.82, 0.85)	28248726.06	0.51	1 (reference)	

Yes	514029	3873	0.75 (0.73, 0.78)	8474788.69	0.46	0.97 (0.94, 1.01)	0.140

Multivariable model was sex, age, body mass index, income levels, smoking, alcohol consumption, regular physical activity, hypertension, diabetes mellitus, dyslipidemia, atrial fibrillation, cancer, renal disease, antiphospholipid syndrome, osteoporotic fracture.

CI, confidence interval; HR, hazard ratio.

Supplementary Table S6. The risk for occurrence of other venous thromboembolism according to oral health status and oral hygiene behaviors.

	Number of participants	Number of events	Event rate (%) (95% CI)	Person-years	Incidence rate (per 1000 person-years)	Adjusted HR (95% CI)	p-value
Oral health status							
Periodontitis							
No	2184950	16160	0.74 (0.73, 0.75)	35740323.51	0.45	1 (reference)	
Yes	61720	669	1.08 (1.00, 1.17)	989735.47	0.68	1.20 (1.11, 1.30)	<.001
Number of missing teeth							
0	1847223	12276	0.66 (0.65, 0.68)	30373054.66	0.40	1 (reference)	
1-7	365761	3941	1.08 (1.04, 1.11)	5887960.71	0.67	1.37 (1.32, 1.42)	<.001
8-14	19586	338	1.73 (1.54, 1.91)	285720.40	1.18	1.51 (1.35, 1.69)	<.001
≥15	14100	274	1.94 (1.71, 2.17)	183323.22	1.50	1.58 (1.40, 1.79)	<.001
Oral hygiene behaviors							
Frequency of tooth brushing (times/day)							
0-1	279192	2867	1.03 (0.99, 1.06)	4431370.27	0.65	1 (reference)	
2	1041889	8678	0.83 (0.82, 0.85)	17023435.58	0.51	0.89 (0.86, 0.93)	<.001
≥3	925589	5284	0.57 (0.56, 0.59)	15275253.14	0.35	0.72 (0.69, 0.75)	<.001
Dental visit for any reason							
No	1348012	9964	0.74 (0.72, 0.75)	21982869.81	0.45	1 (reference)	
Yes	898658	6865	0.76 (0.75, 0.78)	14747189.18	0.47	1.03 (1.00, 1.07)	0.039
Dental scaling							
No	1732641	13312	0.77 (0.76, 0.78)	28253621.34	0.47	1 (reference)	

Yes	514029	3517	0.68 (0.66, 0.71)	8476437.64	0.42	0.96 (0.93, 1.00)	0.050

Multivariable model was sex, age, body mass index, income levels, smoking, alcohol consumption, regular physical activity, hypertension, diabetes mellitus, dyslipidemia, atrial fibrillation, cancer, renal disease, antiphospholipid syndrome, osteoporotic fracture.

CI, confidence interval; HR, hazard ratio.
